# Correction: Boswellic Acid Suppresses Growth and Metastasis of Human Pancreatic Tumors in an Orthotopic Nude Mouse Model through Modulation of Multiple Targets

**DOI:** 10.1371/annotation/d4645c95-739f-4175-b753-b6fe0ba6063c

**Published:** 2012-08-08

**Authors:** Byoungduck Park, Sahdeo Prasad, Vivek Yadav, Bokyung Sung, Bharat B. Aggarwal

The protein extracts derived from cells treated with different concentrations of AKBA (0, 10, 25 and 50 µmol/L) were analyzed by gel electrophoresis. Each sample was electrophoresed in triplicate. At the end of electrophoresis, the proteins were transferred onto the nitrocellulose membrane and membrane was sliced vertically into three pieces:

-Slice # 1: The membrane was sliced horizontally into two pieces; one probed for Bcl-2 and the other for survivin. The blot for Bcl-2 was stripped and reprobed for Bcl-xL.

-Slice # 2: The membrane was probed for c-Myc, then stripped and reprobed first for cyclin D1, and then with COX-2.

-Slice # 3: The membrane was sliced horizontally into two pieces, one was probed for MMP9 and the other for CXCR4. The latter was stripped and reprobed for *-actin.

Since all the samples were run in the same gel, only one *-actin was used.

A revised version of Figure 1C can be viewed here: 

**Figure:**
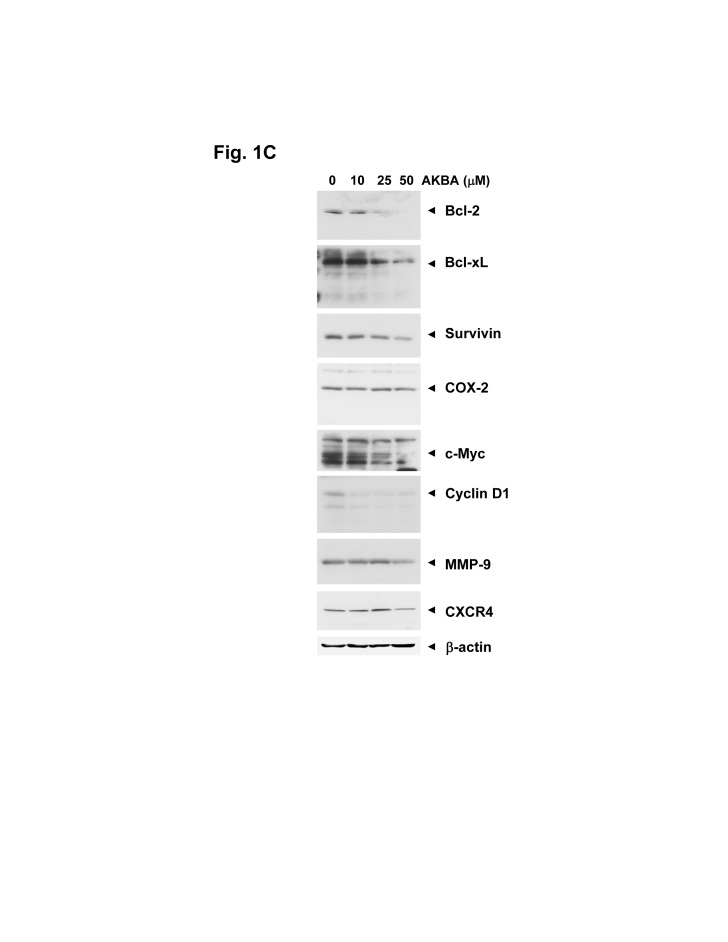


[^] 

